# Non-Biological Slaughterhouse Wastewater Treatment with Membrane Processes—An Opportunity for Water Recycling

**DOI:** 10.3390/nano12132314

**Published:** 2022-07-05

**Authors:** Maximilian Philipp, Jascha Reich, Sven-Uwe Geißen

**Affiliations:** Institut für Technischen Umweltschutz, Fachgebiet Umweltverfahrenstechnik, Technische Universität Berlin, Sekr. KF 2, Straße des 17. Juni 135, 10623 Berlin, Germany; jascha.reich@tu-berlin.de

**Keywords:** non-biological treatment, membrane separation technology, slaughterhouse wastewater, recycling, wastewater treatment, reverse osmosis, ultrafiltration

## Abstract

The pressure-driven membrane separation processes ultrafiltration (UF) and reverse osmosis (RO) enable the effective purification of wastewater, in particular in combination, allowing organic and inorganic contaminants to be separated from the wastewater. Consequently, this work investigates the suitability of this technology for slaughterhouse wastewater (SWW) recycling. This was investigated by means of laboratory and bench-scale plant membrane experiments, whereby slaughterhouse wastewater (SWW) pre-treated by flotation was first treated with UF and then further purified with RO. Through the process combination UF + RO in the bench scale experiment, a reduction of the parameters total organic carbon (TOC), chemical oxygen demand (COD) of more than 98% and 97% for the parameter total nitrogen (TN) could be achieved. This means that wastewater reuse without product contact can be guaranteed. For direct process water reuse, only the concentration limit for ammonium could not be reached. In addition, scanning electron microscopy (SEM) images and energy dispersive X-ray spectroscopy (EDX) analyses of the RO membrane were carried out before and after the experiment, which did not indicate any scaling effects.

## 1. Introduction

In light of the increasing growth of the global population and the accompanying rise in demand for water [1,2], recycling industrial wastewater appears to be a suitable way of water conservation [3]. Membrane treatment has proven to be an efficient and well-suited technology in the field of wastewater reuse due to its high operational reliability, considerable recovery rate and high permeate water quality [3,4,5]. Since slaughterhouses are responsible for a large proportion of water consumption in the food processing industry [6], the reuse of SWW by means of membrane separation were investigated. The composition of SWW varies considerably depending on the different processes and the specific water demand [6]. SWW is considered harmful worldwide due to its complex composition of fats, proteins and fibres, as well as the pathogenic risk posed by faecal bacteria and possible infectious carcasses [6,7]. Additionally, SWW has a comparatively high N/C ratio [6,8], which might inhibit anaerobic processes and lead to an elaborated biological nitrogen elimination process. Therefore, the focus of this work was placed on the chemical-physical treatment. In the past, these processes have proven to be an effective technology with high operational reliability in the field of SWW treatment [6,8]. Starting with flotation, which has been proven to be the most effective pre-treatment method for heavily loaded SWW in most cases, an additional membrane-based technology is to be examined [9]. This novel method has become widely adopted by researchers due to its enormous advantages over conventional biological processes [4]. The combination of processes used in this work are in accordance with Racar et al. [10] and Coskun et al. [11]. In these publications, it has been found that, after appropriate pre-treatment, it is possible to produce water for reuse by treating SWW with RO [10,11].

In the work conducted by Racar et al. [10] the sequencing batch reactor (SBR)-sand filter-UF-nanofiltration (NF)/RO processes were combined. Thereby, a treatment of SWW could be achieved, which allows a reuse for applications without product contact (e.g., external rinsing and cleaning processes or boiler feed water). Furthermore the work of Coskun et al. [11] also examines the treatment of SWW. The combinations of centrifuge-UF-NF/RO indicated good results for the SWW reuse. However, the nitrogen concentrations of the treated SWW samples and the produced permeate were not specified, therefore it cannot be conclusively clarified whether a reuse of process water is possible. Since RO achieved a higher purification performance than NF in both studies, it was investigated in this study.

With regard to possible targets for the potential use of treated wastewater, the focus was on the possibilities of direct process water reuse and water reuse without direct product contact. In this context, the European Drinking Water Regulation and the EU Regulation on minimum requirements for water reuse should be used as a reference for the assessment [12,13]. The resulting concentration limits can be found in Table 1.

## 2. Materials and Methods

### 2.1. Slaughterhouse Wastewater

SWW samples from two slaughterhouses with different capacities were examined: A small rural slaughterhouse with a slaughter capacity of about 1 cattle per day and a wastewater production of 1 m^3^ per day and a large slaughterhouse with a slaughter capacity of about 60,000 chickens per day and and a wastewater production of 600 m^3^ per day. The wastewater from the large slaughterhouses comes from the mixing and equalization basins, in which the wastewater from all processes in the slaughterhouse is collected. This includes: decapitation, scalding, defeathering and evisceration, as well as the wash water from the building, vehicle and bleeding basin cleaning. However, the main part of the blood is disposed separately. The wastewater from the small slaughterhouse is from the effluent of the grease separator, in which all the wastewater from the slaughterhouse, including the blood and the domestic waste water from the butchery, is collected. Both are characterized and compared with reference values from the “Reference Document on Best Available Techniques in the Slaughterhouses and Animal By-products Industries, 2005" BREF [9] in Table 2.

For the membrane separation experiments only pre-treated SWW was used. The larger slaughterhouse operates a dissolved air flotation (DAF) on site. These samples were taken from the purified flow of the DAF. The samples from the small slaughterhouse were taken after the fat separator at different times and refrigerated at 4 °C until analysis. These samples were prepared by flocculation using 600 mg/L ferric chloride as well as 10 mg/L of flocculant AF 1245. Afterwards they were filtered through a fabric to separate all flocs. The characterisation of both pre-treated samples are shown in Table 3.

### 2.2. Analytical Methods

To characterize SWW with regard to possible treatment options, the following parameters were analyzed: pH, conductivity, COD, biological oxygen demand (BOD5), TOC, dissolved organic carbon (DOC), TN and total suspended solids (TSS). The pH value was determined with a METTLER TOLEDO pH meter. The mobile conductivity meter Cond 340i from the company WTW was used for the conductivity measurements. The COD was measured by means of the QuickCOD_lab_-03D0318 from the company LAR Process Analysers AG via a thermal disintegration process. BOD5 was measured by pressure determination with a WTW Oxitop Control 12 system (Weinheim, Germany). The measurement of TOC, DOC and TN was performed with the Analytik Jena TOC analyzer multi N/C 3100 (Jena, Germany), whereby the DOC samples were prepared by filtration through Whatman 0.45 µm membrane filters (Kent, UK).

In addition, the flat sheet membranes used were analysed by using conventional SEM using a Zeiss DSM 982 GEMINI microscope with a thermal field emission cathode optimised for high resolution and X-ray analyses at low accelerating voltage. This has the following specifications: Inlens secondary electron detector, Everhart-Thornley chamber secondary electron detector, K.E.D. 4-quadrant BSE detector, point electronic imaging system, EDAX EDX-system (Apollo XPP with nom. 10 mm^2^ (SDD), energy resolution 123.9 eV @ Mn-Kα).

### 2.3. Experimental Setup

**Figure 1:**
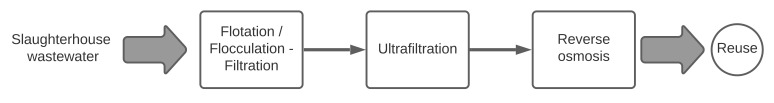
Process flow diagram.

Due to the poor water solubility of the organic components in SWW and the high fat content, flotation is recommended as the first treatment step [8,9]. Therefore, the process combination of DAF–UF–RO was investigated and evaluated with respect to its suitability for SWW reuse. The process is shown in Figure 1.

A particular challenge in membrane filtration is the blocking of the membrane by fouling and scaling [14]. Therefore, special attention was given to the flux reduction during longer operation periods of the UF-system. In these investigations, membrane experiments were initially carried out on a laboratory scale. Subsequently, an upscaling and longer operation were investigated with bench-scale plants. Special attention was given to the UF, which has already been investigated for the treatment of SWW [15,16]. Finally, the influence of cross-flow velocity and transmembrane pressure (TMP) on flux and permeate quality were investigated in a long-term test. Four experiments were carried out in total. One preliminary experiment and one experiment at a bench-scale were carried out with UF and RO, respectively. An overview of the experiments is given in Table 4.

#### 2.3.1. Ultrafiltration Small-Scale Experiments

**Figure 2:**
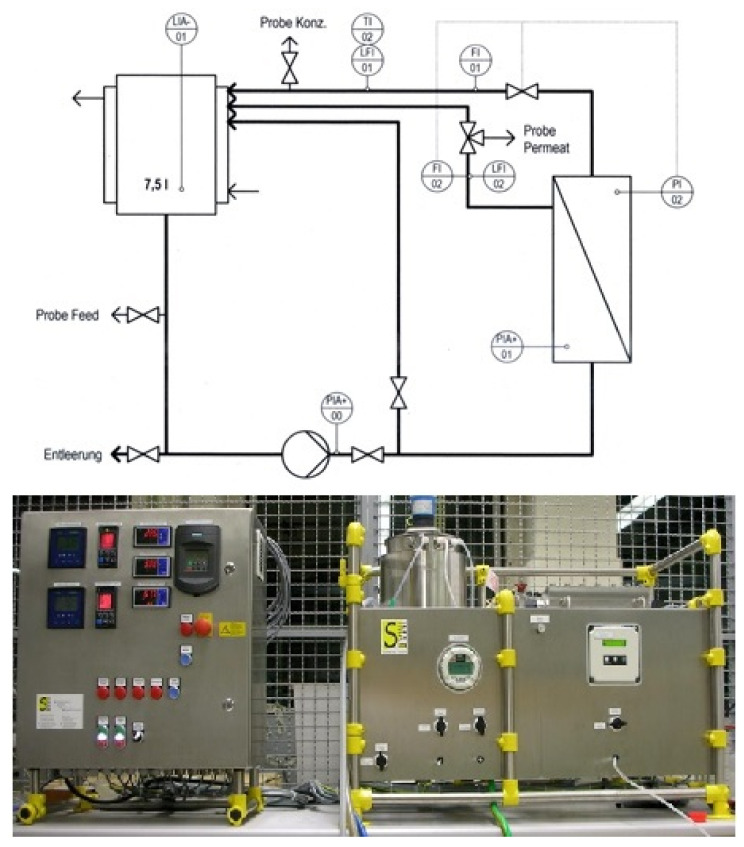
Flat sheet membrane test system LSta80.

In order to find a suitable UF membrane, preliminary tests were carried out. For the experiments of the UF and RO flatsheet membranes, the laboratory membrane test system LSta80 from SIMA-tec (41366 Schwalmtal, Germany) was used. The flow diagram of the plant is shown in Figure 2. The plant can be used for investigations in the field of microfiltration, UF, nanofiltration and RO and can be operated with a pressure of up to 100 bar. Organic flat film membranes with an active membrane area of 86 cm^2^ were used. The system has a double-walled feed tank with a capacity of 7.5 L, which was kept at 20 °C by a temperature regulator. Manual valves allow samples to be taken from the feed, concentrate and permeate streams during operation. The readings for TMP, flow rate, electrical conductivity and pH were taken continuously and a data point were recorded every third second.

For the UF experiments with the LSta80 membrane test system, a UH030 polyethersulfone membrane from Mann + Hummel with a molecular cut-off size of 30 kDa was used. In these experiments, permeate was continuously withdrawn after a start-up phase of 15 min until a yield of 80% was achieved. An SEM image of the flat sheet UH030 membrane used in the experiment and a comparable membrane used with the bench-scale experiment described in Section 2.3.2 can be found in Appendix A.1.

#### 2.3.2. Ultrafiltration Bench-Scale Plant, Long-Term Experiment

**Figure 3:**
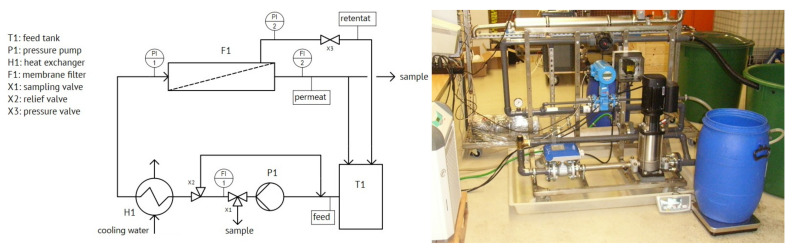
Ultrafiltration bench-scale plant.

The preliminary tests with the UF showed that not the pore size, but the cross-flow velocity and the TMP were most critical for the flux and the filter performance. Therefore, a long-term test was conducted to determine the operating conditions favourable for a low-maintenance UF treatment. A bench-scale plant was used for these tests and pre-treated SWW was used. The plant shown in Figure 3 allows an operating pressure of up to 10 bar and achieves a feed volume flow of up to 5.7 m^3^·h^−1^ by means of an adjustable pressure pump. The volume flows in the feed and in the permeate outlet are recorded by means of inductive flow meters. The pressure can be measured by means of a pressure gauge upstream and downstream of the membrane. The storage tank has a maximum capacity of 300 L. For the tests, a M-C32-08-1200-0.20-VFU100-PVC-U 7-channel tubular membrane with a membrane area of 0.2 m^2^ and a molecular cut-off size of 100 kDa was used.

In these experiments, pre-treated SWW was circulated at different TMPs and cross-flow velocities, to identify the optimum operating conditions. To inhibit bacterial degradation of the contained organics, 0.3 g·L^−1^ of ClO_2_ was added to the sample. Different TMPs were first applied at a constant cross-flow velocity of 4 m·s^−1^ until a constant permeate volume flow was achieved over several days. The pressure was increased from 0.5 bar in steps of 0.5 bar up to a TMP of 2 bar. After the ideal TMP was determined, the velocity was lowered. With a constant TMP of 1.5 bar, the cross-flow velocity was first reduced to 2 m·s^−1^, then increased to 3 m·s^−1^ and finally set to 4 m·s^−1^ again. When the flux dropped to 60 L·h^−1^·m^2^, the plant was emptied and the membrane was washed with a 30 °C warm 1.5% Ultrasil^®^53 solution for 3 h. The solution was then drained and the plant rinsed with tap water until no more foaming was detected. New feed was then added to the system and the experiment continued.

#### 2.3.3. Reverse Osmosis Small-Scale Experiments

For the reverse osmosis pre-tests, the laboratory membrane test system LSta80, as described in Section 2.3.1 was used. In this experiment, the polyacrylonitrile membrane BW30 was applied. The sample used was SWW, which was pretreated by DAF and UF as described in Chapter 2.3.1. In the experiment, the TMP was kept at 20 bar and permeate was withdrawn until a yield of 80% was achieved.

#### 2.3.4. Reverse Osmosis Bench-Scale Plant

**Figure 4:**
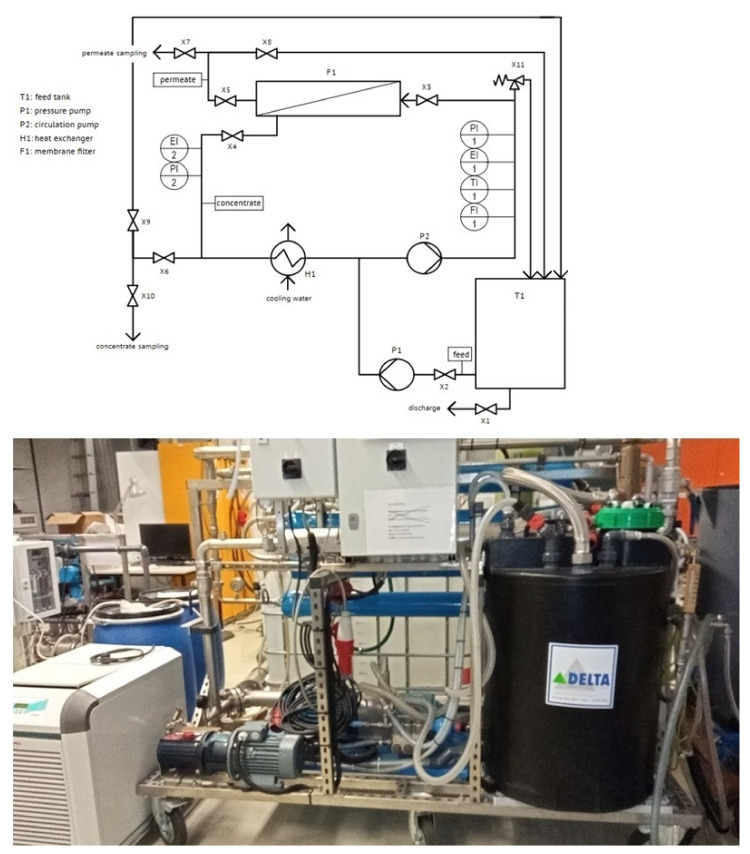
Reverse osmosis bench-scale plant.

In order to determine the optimum operating conditions in terms of TMP and permeate yield for the RO, the bench-scale plant shown in Figure 4 with a FilmTec™ BW30-4040 membrane module was used. SWW pre-treated with UF was used in the experiment. During the experiment, SWW was circulated at different TMPs of 10, 15 and 20 bar. The TMP levels were maintained for one hour and then permate was withdrawn until a yield of 50% and 75 was achieved, respectively.

## 3. Results and Discussions

### 3.1. Ultrafiltration Preliminary Experiments

**Figure 5:**
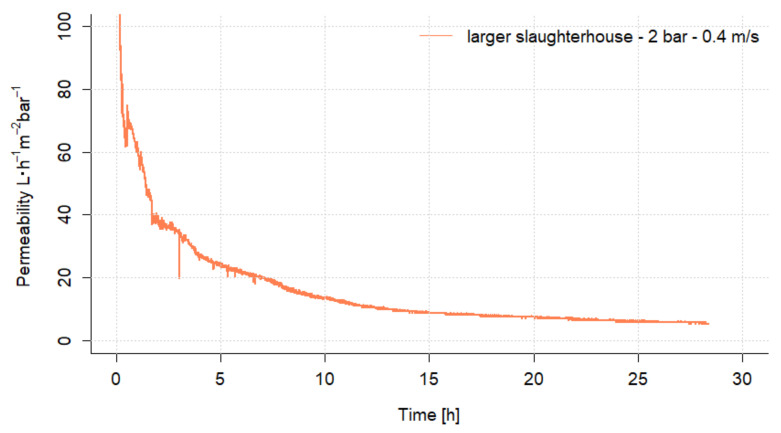
Permeability for ultrafiltration with flat sheet membrane 30 kDa.

The permeability and the rejection of water impurities were investigated in the first experiment with the UF flat sheet membrane. The TMP was kept constant at 2 bar and the permeate was continuously withdrawn until a yield of 80% was achieved. The permeability trend versus time is shown in Figure 5. It can be seen that the permeability decreases strongly with time and drops from initially over 100 to 5.2 L·h^−1^·m^−2^·bar^−1^ by the end of the experiment.

In the experiment TOC, TN and COD were measured in the feed and permeate. The mean retention of the SWW components is shown in Table 5. A medium rejection was achieved for the COD and a low rejection for TOC and TN. As further preliminary tests had shown: No significantly higher rejection could be achieved with a smaller cut-off size. However, a NF membrane was not applied due the much smaller flux. Since further preliminary tests, as well as the work of Malmali et al. [15], show that the retention is comparable up to a molecular separation size of 100 kDA, but that the transmembrane flux increases with larger pore size, a membrane with a molecular separation size of 100 kDa was chosen for the following experiments with the bench-scale plant.

### 3.2. Ultrafiltration Bench-Scale Plant, Long-Term Experiment

In these experiments pre-treated SWW characterised in Table 3 was used as feed. This was circulated in the experiments and only occasionally removed and filled with fresh samples. Despite the use of biocides, the COD and TOC concentrations in the feed decreased slowly. When the COD concentration droped below 350 mg·L^−1^, the feed was renewed with fresh pre-treated SWW.

**Figure 6:**
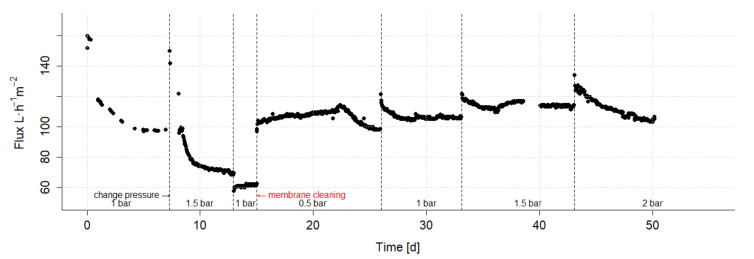
Long-term ultrafiltration experiment: Flux in dependence of transmembrane pressure at a constant cross-flow velocity of 4 m·s^−1^.

The influence of the TMP on the transmembrane flux at a constant cross-flow velocity of 4 m·s^−1^ was investigated in the first long-term experiment. The change in flux at different TMP levels is shown in Figure 6. At the beginning of the experiment, within the first days, a decrease of the flux from 160 to 100 L·h^−1^·m^2^ is observed, this is a repeatedly observed effect when a membrane is fed with real wastewater for the first time. However, the flux of 100 L·h^−1^·m^2^ can still be considered high in comparison [15]. After 15 days, the flux decreased from an initial 160 L·h^−1^·m^2^ to the lowest value of 60 L·h^−1^·m^2^. When this value was reached, the plant was emptied and the membrane was cleaned once with Ultrasil^®^53. The feed tank was then refilled and the sample continued to be circulated. Subsequently, a more stable flux was observed. Immediately after membrane cleaning, a flux of 100 L·h^−1^·m^2^ was observed at a TMP of 0.5 bar. In addition, the flux subsequently increased to 115 L·h^−1^·m^2^ over 7 days, only to drop again to 97 L·h^−1^·m^2^ after 3 days at the same TMP. at this point in the experiment, it was intended to check the extent to which the preformed cover layer is reversible. It could be shown that this is partly true. It could also be observed that under the conditions of high overflow velocity and low pressure at the beginning, the cover layer can be sheared off well in the short term due to the high velocity, which results in an increase in flux, but after a longer period of time it is slowly built up again, which leads to a renewed decrease in flux. At 1.5 bar the flux was as high as approximately 110 L·m^−2^·h^−1^ and stable over a longer period of time. Therefore, 1.5 bar was chosen for the later experiments.

**Figure 7:**
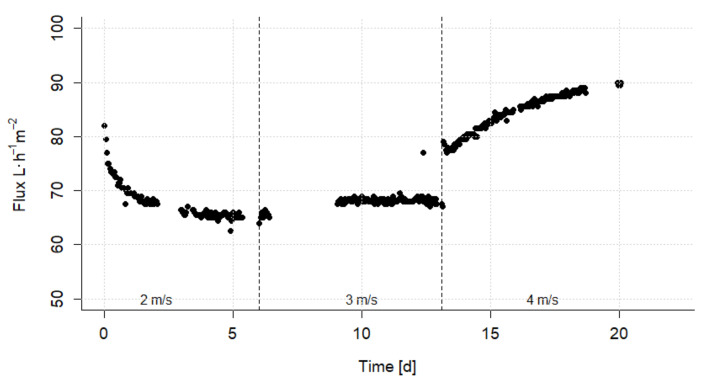
Long-term ultrafiltration experiment: Flux in dependence of the cross-flow velocity at a constant pressure of 1.5 bar.

In the second phase of the experiments, the influence of the cross-flow velocity on the flux at a constant pressure of 1.5 bar was investigated. The results are shown in Figure 7. The cross-flow velocity of 2 m·s^−1^ led to a strong decrease of the flux to 65 L·h^−1^·m^2^. By increasing the cross-flow velocity to 3 m·s^−1^, the flux improves only slightly. The further increase to 4 m·s^−1^ resulted in a rising flux up to 90 L·h^−1^·m^2^. Thus, it can be observed that the change in the surface layer can be controlled by adjusting the cross-flow velocity and that this effect is partially reversible.

**Figure 8:**
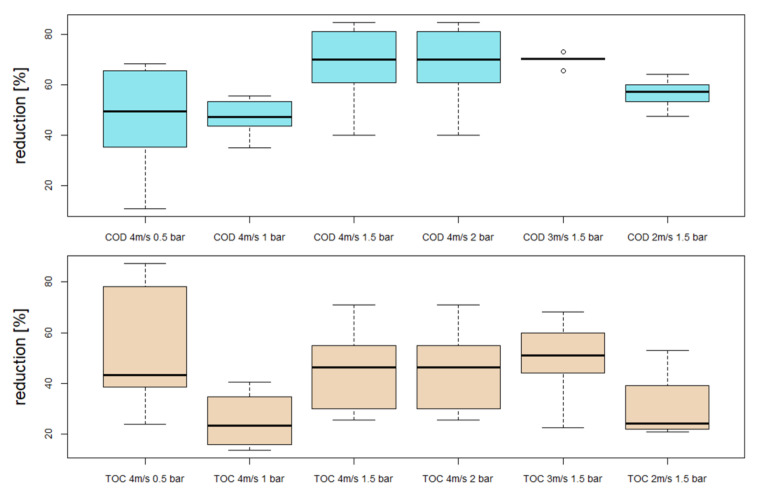
Rejection of COD and TOC in dependence of cross-flow velocity and transmembrane pressure.

With regard to the rejection rates of the membrane under different operating conditions, it was observed that for the parameters TN and turbidity, the reduction was comparatively constant for all experiments at 10% and 97%, respectively. However, significant fluctuations were observed for the parameters COD and TOC. The reduction of the parameters COD and TOC at the different operating conditions are shown in Figure 8, only samples taken during stable operation, defined by small fluctuations in flux, were considered. On average, the highest rejection rates were achieved at 1.5 and 2 bar. Consequently, the best overall performance was achieved with a TMP of 1.5 bar and a cross-flow velocity of 3 m·s^−1^. It can be observed that increasing TMP at the same cross-flow velocity leads to a compressed surface layer which seems to reject COD and TOC better. However, with 3 m·s^−1^ the transport through the layer is reduced. Similar results could also be observed in the work of Boyle-Gotla et al. [17] and Thomassen et al. [18].

### 3.3. Reverse Osmosis Small-Scale Experiments

**Figure 9:**
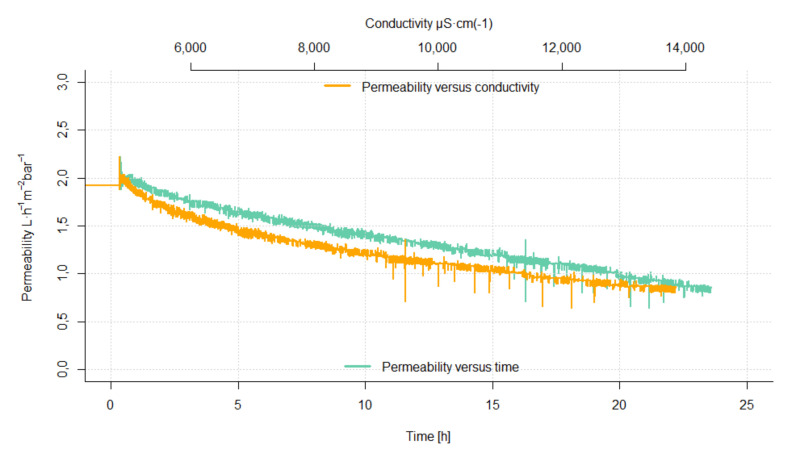
Permeability for reverse osmosis with flat sheet membrane BW30 at 20 bar.

**Figure 10:**
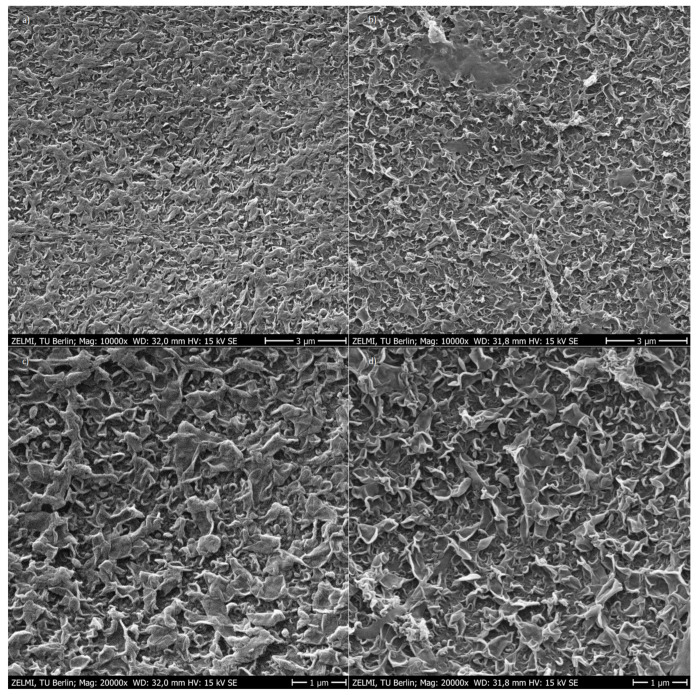
(**a**) BW30 flat sheet membrane unused 10,000× magnification, (**b**) BW30 flat sheet membrane used 10,000× magnification, (**c**) BW30 flat sheet membrane unused 20,000× magnification, (**d**) BW30 flat sheet membrane used 20,000× magnification.

**Figure 11:**
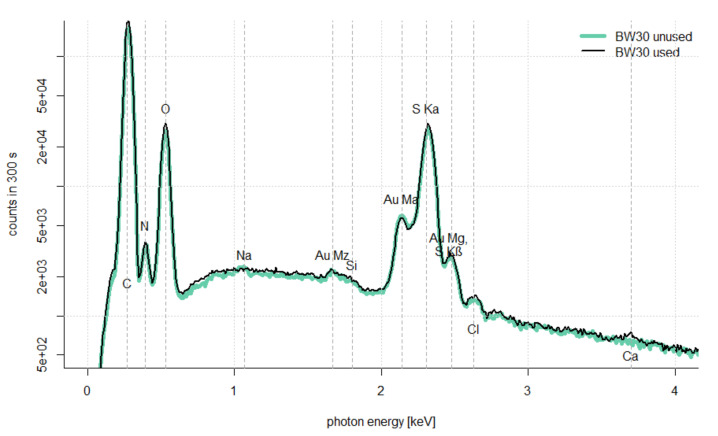
Energy dispersive radiography spectroscopy of sheet membrane BW30 before and after experiment.

In order to investigate the TMP and the rejection of water impurities by the RO, an experiment with the membrane test cell was carried out. The permeate from the UF experiment described in Section 2.3.1 was used as feed. Figure 9 shows the permeability over time of a BW30 flat sheet membrane under continuous permeate withdrawal, as well as permeability versus electrical conductivity. A decrease in permeability from 2 to 0.8 L·h^−1^·m^−2^·bar^−1^ was observed, this can most likely be attributed to the increasing salinity in the feed and does not necessarily translate into a blockage of the membrane, in order to investigate this in more detail further tests were carried out with the membrane. Figure 10 shows SEM images of the flat sheet membrane used before and after the experiment. No optical change of the membrane could be identified. In addition, EDX was performed on the membrane before and after the experiment. The results are shown in Figure 11. It can be observed that the elemental composition of the used and unused membrane is not significantly different, solely the peak for sodium and calcium could be discussed. Based on this data, no indication of scaling effects could be found over the duration of the experiment.

Table 6 gives the feed, permeate, and retentate values of the TOC, TN, COD and conductivity as well as the rejection. A very high rejection of more than 94% for all investigated parameters was observed. The values measured in the permeate meet both the limits for process water reuse and for reuse without product contact from Table 1.

### 3.4. Reverse Osmosis Bench-Scale Plant Experiments

**Figure 12:**
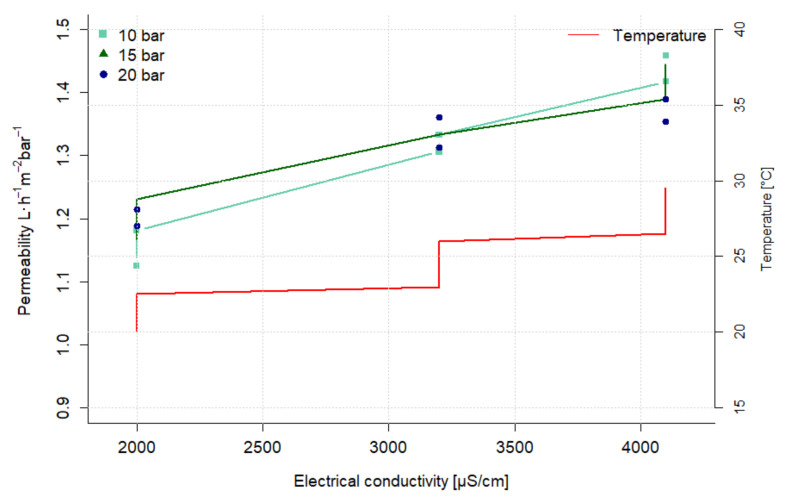
Permeability as a function of conductivity for reverse osmosis with BW30 membrane at different transmembrane pressures.

The feed for this experiment was the permeate produced with the long-term UF experiments described in Section 2.3.2. It should be noted that the concentrations of the impurities are slightly lower because of the dilution by the residual water in the plant at the beginning of the experiment. The permeability as a function of the electrical conductivity for the various TMPs is shown in Figure 12. The permeability increases at all pressure levels with the electrical conductivity from 1.2 L·h^−1^·m^−2^·bar^−1^ to 1.4 L·h^−1^·m^−2^·bar^−1^. However, this can be explained by the increasing temperature. Dividing the permeability by the respective temperature gives a normalized permeability of 0.06 L·h^−1^·m^−2^·bar^−1^·°C^−1^ at the beginning of the experiment (2000 µS·cm^−1^) and 0.05 L·h^−1^·m^−2^·bar^−1^·°C^−1^ at the end of the experiment (4000 µS·cm^−1^). So the normalized permeability decreases with increasing electrical conductivity, which can be explained by the rising osmotic pressure. Consequently, no scaling effects can be observed over a period of 24 h.

Similar to the preliminary concentrating experiment, a rejection of more than 95% could be observed for all analyzed parameters. The characterisation of the feed, permeate and retentate, as well as the resulting retention rates, are shown in Table 7. All limits for reuse without product contact summarized in Table 1 can be met. For direct process water reuse, however, the concentration for ammonium with 3 mg·L^−1^ is above the limit of 0.5 mg·L^−1^.

## 4. Conclusions

In the experiments with the UF, it was observed that the membrane pore size has a smaller influence on retention and permeability than the surface layer. Furthermore, a direct influence between the operating parameters TMP and cross-flow velocity on the surface layer and the associated permeability and retention could be shown. It was observed that UF is an effective way to treat partial flows of SWW as well as pre-treated SWW. In this way, the SWW can be prepared for possible further treatment by RO. The optimal operating conditions for the treatment of pre-treated SWW, are a TMP of 1.5 bar and a cross-flow velocity of 4 m·s^−1^ for the PVC UF membrane used. Thus, a very high and stable flux of over 90 L·h^−1^·m^2^ could be achieved.

The use of RO as a polishing step in the treatment of SWW was also demonstrated to be effective here. A stable permeability of more than 1 L·h^−1^·m^−2^·bar^−1^ could be measured when the concentration was increased to an electrical conductivity of more than 9 m·S·cm^−1^. The BW30 RO membrane used was examined with SEM before and after use and the surface was analysed with EDX. Even after elevating the electrical conductivity to high values, no indications of scaling effects could be found within the scope of the experiment. Therefore, it can be assumed that an even higher concentration and a corresponding higher water yield can be achieved with the RO. Nonetheless, a total water yield of 60% was achieved with the process combination investigated.

With regard to the reusability of the purified SWW, the process combination DAF-UF-RO can achieve reuse without product contact, since a complete reduction of microbial contaminants can be guaranteed by the multi-barrier principle containing both UF and RO in all cases. Therefore, the permeate produced can safely be used for the cleaning of buildings and vehicles as well as similar applications. The direct reuse of the permeate as process water complies with most of the concentration limits that apply to drinking water in the EU. However, the concentration limit for COD could only barely be achieved. Furthermore, the concentration limit for ammonium of 0.5 mg·L^−1^ could not be reached in this work, as the values registered were 3 mg·L^−1^ and higher. Therefore, a further process step for the reduction of COD and ammonium should be added to allow for direct process water reuse.

## Figures and Tables

**Table 1:** Limits for wastewater reuse.

Parameter	Unit	Process Water Reuse [12]	Reuse without
		(Drinking Water Quality)	Product Contact [13]
BOD_5_	[mg·L^−1^]	<1	-
COD	[mg·L^−1^]	5	-
Nitrate	[mg·L^−1^]	50	-
TOC	[mg·L^−1^]	No abnormal change	-
*E. coli*.	(cfu· 1 L^−1^)	0	0
*Legionella* spp.	(cfu· L^−1^)	-	<1000
Turbidity	[NTU]	10	5
Conductivity	[µS·cm^−1^]	2500	-
Na^+^	[mg·L^−1^]	200	-
NH_4_^+^	[mg·L^−1^]	0.5	-
F^−^	[mg·L^−1^]	1.5	-
Cl^−^	[mg·L^−1^]	250	-
NO_3_^−^	[mg·L^−1^]	50	-
SO_4_^2−^	[mg·L^−1^]	250	-

**Table 2:** Slaughterhouse wastewater characteristics.

Parameter	Unit	Small Slaughterhouse	Large Slaughterhouse	BREF [9]
TOC	[mg·L^−1^]	700–900	900	
TN	[mg·L^−1^]	250–440	260	40–300
COD	[mg·L^−1^]	2000–3000	400	1000–5000
BOD_5_	[mg·L^−1^]	600–800		500–2500
TP	[mg·L^−1^]	70–90	75	
TSS	[mg·L^−1^]	1600–1900	2600	1000–2000
PH		7.5–8.3	7.6	6.0–9.0
Conductivity	[µS·cm^−1^]	2500–3300	3100	

**Table 3:** Pretreated slaughterhouse wastewater.

Parameter	Unit	Small Slaughterhouse	Large Slaughterhouse
TOC	[mg·L^−1^]	250–360	360
TN	[mg·L^−1^]	100–200	140
COD	[mg·L^−1^]	400–1100	1080
TSS	[mg·L^−1^]	150–300	260
Conductivity	[µS·cm^−1^]	2800–4000	3600

**Table 4:** Overview of the performed experiments.

Experiment	Membrane	Module	Comment
ultrafiltration	UH030 PES-30 kDa	flat sheet	permeate withdrawal
small scale experiments	Mann + Hummel	LSta80	up to 80% yield
ultrafiltration	M-C32-08-1200	tubular 0.2 m^2^	permeate circulated
bench-plant	VFU 100 kDA-PVC-U	7-channel	Long-term experiment
reverse osmosis	BW30	flat sheet	permeate withdrawal
small scale experiments	polyacrylonitrile	LSta80	up to 80% yield
reverse osmosis	BW30-4040	spiral wound	permeate circulated
bench-plant	FilmTec™	7.2 m ^2^	at different TMP levels

**Table 5:** Rejection by means of UH030 flat sheet membrane at 80% yield.

Parameter	Unit	Feed	Permeate	Reduction [%]
TOC	[mg·L^−1^]	180	150	15
TN	[mg·L^−1^]	60	50	18
COD	[mg·L^−1^]	475	210	55

**Table 6:** Rejection of the examined parameters by means of BW30 flat sheet membrane at 80% yield.

Parameter	Unit	Feed	Permeate	Retentate	Reduction [%]
TOC	[mg·L^−1^]	280	3	1400	99
TN	[mg·L^−1^]	80	5	225	94
COD	[mg·L^−1^]	500	3	2,500	99
Conductivity	[µS·cm^−1^]	3500	110	8700	97

**Table 7:** Rejection of the examined parameters by means of a BW30 spiral wound membrane module at 75% yield.

Parameter	Unit	Feed	Permeate	Retentate	Reduction [%]
COD	[mg·L^−1^]	135	4	260	97
TN	[mg·L^−1^]	80	3	180	96
TOC	[mg·L^−1^]	25	<0.5	45	>98
Conductivity	[µS·cm^−1^]	2000	50	4,000	98
Na^+^	[mg·L^−1^]	150	2	270	99
NH_4_^+^	[mg·L^−1^]	65	3	120	95
k^+^	[mg·L^−1^]	90	1	160	99
Ca^2+^	[mg·L^−1^]	70	<0.5	250	>99
Mg^2+^	[mg·L^−1^]	15	<0.5	30	>97
F^−^	[mg·L^−1^]	<0.5	<0.5	2	-
Cl^−^	[mg·L^−1^]	270	1	460	99
NO_3_^−^	[mg·L^−1^]	5	<0.5	12	>99
SO_4_^2−^	[mg·L^−1^]	55	<0.5	80	>99
PO_4_^3−^	[mg·L^−1^]	15	<0.5	30	>99

**Figure A1:**
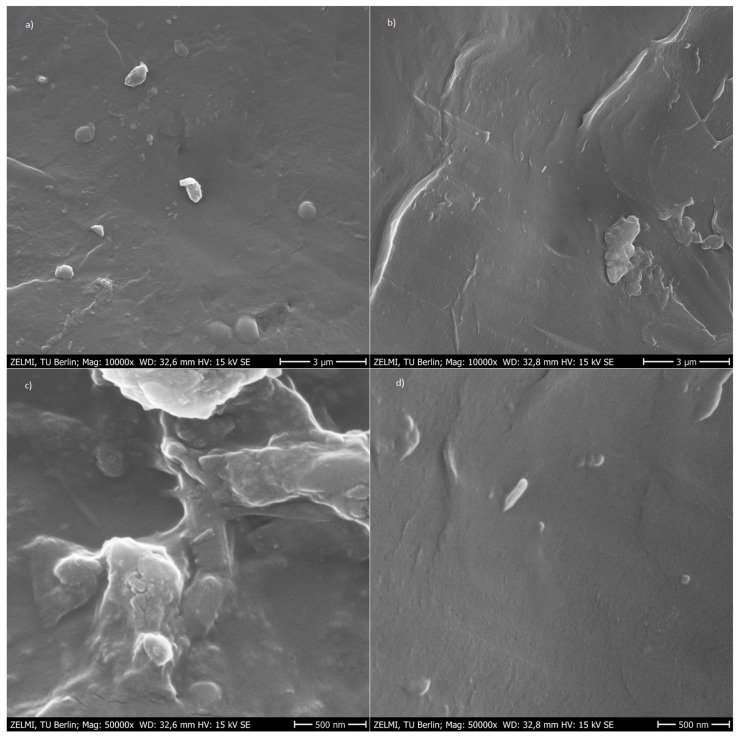
(**a**) ultrafiltration flat sheet membrane 100 kDa 10,000× magnification, (**b**) ultrafiltration flat sheet membrane 30 kDa 10,000× magnification, (**c**) ultrafiltration flat sheet membrane 100 kDa 50,000× magnification, (**d**) ultrafiltration flat sheet membrane 30 kDa 50,000× magnification.

## Data Availability

Data presented in this article are available at request from the corresponding author.

## Abbreviations

BOD_5_	biological oxygen demand
COD	chemical oxygen demand
DAF	dissolved air flotation
DOC	dissolved organic carbon
EDX	energy dispersive X-ray spectroscopy
NF	nanofiltration
RO	reverse osmosis
SBR	sequencing batch reactor
SEM	scanning electron microscopy
SWW	slaughterhouse wastewater
TMP	transmembrane pressure
TN	total nitrogen
TOC	total organic carbon
TSS	total suspended solids
UF	ultrafiltration
